# Exploring knowledge valorization and utilization strategies in academic health science centers in the Netherlands: multi‐case study of Alzheimer centers

**DOI:** 10.1002/alz.088138

**Published:** 2025-01-09

**Authors:** Eden Meng Zhu, Martina Buljac‐Samardžić, Kees Ahaus, Robbert Huijsman

**Affiliations:** ^1^ Erasmus University Rotterdam, Rotterdam Netherlands

## Abstract

**Background:**

‘Intellectual assets’ generated in traditional university settings, that may not fit the interests of the standard ‘valuation criteria’ (i.e. commercially profitable), such as non‐pharmacological dementia care research, often remain siloed within their respective research disciplines and originating institutions. Implementation science can be leveraged to navigate the complexity of research innovation systems and enhance the outcomes of research valorization and utilization activities. Five specialized Alzheimer Centers were established to facilitate the creation, translation, implementation, and dissemination process for dementia research in the Netherlands. This study aims to use an implementation science‐guided approach to explore the knowledge valorization, implementation and dissemination activities present within the Centers to understand the activities and strategies used at each stage of the ‘knowledge‐to‐action’ process.

**Method:**

Individual semi‐structured qualitative interviews from participants through purposive sampling of researchers employed across five Alzheimer Centers were conducted, and multiple case studies were produced. Interviews were conducted in‐person and virtually through Microsoft Teams, and all were audio‐recorded, transcribed verbatim, and analyzed using ATLAS.ti software. Leaders of each Alzheimer Center were involved in later stages of data analysis to help interpret and validate the results.

**Result:**

Knowledge creation, and the translation of research findings into research products, was facilitated by the proximity between researchers and dementia patients, through ‘memory clinics’ and ‘client panels’ that provided researchers the opportunity to involve end‐users in research product development. Implementation of knowledge products was often facilitated through establishing early formal public‐private partnerships, involving well‐networked external intermediary organizations and university‐based technology transfer offices to determine implementation pathways. Dissemination was facilitated through strategically leveraging social media marketing tactics to expand reach and establishing partnerships to utilize existing dissemination channels.

**Conclusion:**

Although similar knowledge valorization and utilization strategies were identified across the five Alzheimer Centers, the degree of success for the strategies found varied across each Center, which suggests a connection between the Alzheimer Center’s organizational maturity, the implementation teams’ overall readiness, and the selected strategies’ outcomes. Future studies may explore the relationship between organizational characteristics and implementation support readiness to develop a context‐appropriate implementation mapping approach, suitable for enhancing implementation support readiness in dementia research.

